# CULTURALLY ADAPTING A TAILORED ACTIVITY PROGRAM FOR CHINESE OLDER ADULTS LIVING IN NURSING HOMES

**DOI:** 10.1093/geroni/igae098.2447

**Published:** 2024-12-31

**Authors:** Yu Zheng, Wenting Peng, Minhui Liu, Ni Gong, Chunxiao Li

**Affiliations:** The Third Xiangya Hospital of Central South University, Changsha, Hunan, China (People’s Republic); University of Washington, Seattle, Washington, United States; Ningxia Medical University, Yinchuan, Ningxia, China; Central South University, Changsha, Hunan, China (People’s Republic); Central South University, Changsha, Hunan, China (People’s Republic)

## Abstract

The behavioral and psychological symptoms of dementia are aggravating among older adults at nursing homes. However, in China, there is no recognized effective program that has improved the behavioral and psychological symptoms of dementia. Therefore, we aimed to cross-culturally adapt and localize a foreign evidence-based intervention program (Tailored Activity Program, TAP), and examine its feasibility and acceptability. A total of 20 people with dementia and 12 caregivers participated in the program, and after 8 sessions. Results showed that there were significant improvements in behavioral and psychological symptoms, daily living activities, depression of older adults and depression, caregiving burden, caregiving response load of caregivers (P < 0.05), but there were no significant changes in quality of life, cognitive function of older adults and caregivers’ readiness (P > 0.05). In the semi-structured interview section, the interview data of people with dementia could be summarized as improved negative emotions, relieved stress, disinterest in activities, and activity-related suggestions; the interview data of caregivers could be summarized as improved negative emotions, relieved stress, and desire to be understood. Finally, 2 adapted suggestions (extending the duration of interventions and adjusting inclusion-exclusion criteria) were obtained based on the feedback from interview section. In conclusion,18 of 20 people with dementia (90% > 80%) and all of 12 caregivers (100% > 80%) fully understood and were willing to accept the program. This program improves the emotional experience, with a high degree of feasibility and acceptability. The tailored activity program was considered to be adapted and localized.

